# Dynamics of Bacterial Communities in Two Unpolluted Soils after Spiking with Phenanthrene: Soil Type Specific and Common Responders

**DOI:** 10.3389/fmicb.2012.00290

**Published:** 2012-08-21

**Authors:** Guo-Chun Ding, Holger Heuer, Kornelia Smalla

**Affiliations:** ^1^Julius Kühn-Institut, Federal Research Centre for Cultivated PlantsBraunschweig, Germany

**Keywords:** unpolluted soil, phenanthrene, bacterial communities, DGGE, pyrosequencing

## Abstract

Considering their key role for ecosystem processes, it is important to understand the response of microbial communities in unpolluted soils to pollution with polycyclic aromatic hydrocarbons (PAH). Phenanthrene, a model compound for PAH, was spiked to a Cambisol and a Luvisol soil. Total community DNA from phenanthrene-spiked and control soils collected on days 0, 21, and 63 were analyzed based on PCR-amplified 16S rRNA gene fragments. Denaturing gradient gel electrophoresis (DGGE) fingerprints of bacterial communities increasingly deviated with time between spiked and control soils. In taxon specific DGGE, significant responses of *Alphaproteobacteria* and *Actinobacteria* became only detectable after 63 days, while significant effects on *Betaproteobacteria* were detectable in both soils after 21 days. Comparison of the taxonomic distribution of bacteria in spiked and control soils on day 63 as revealed by pyrosequencing indicated soil type specific negative effects of phenanthrene on several taxa, many of them belonging to the *Gamma-*, *Beta-*, or *Deltaproteobacteria*. Bacterial richness and evenness decreased in spiked soils. Despite the significant differences in the bacterial community structure between both soils on day 0, similar genera increased in relative abundance after PAH spiking, especially *Sphingomonas* and *Polaromonas*. However, this did not result in an increased overall similarity of the bacterial communities in both soils.

## Introduction

Anthropogenic activities such as combustion of wood, coal, and petroleum or mining accidents frequently result in the pollution of soils with polycyclic aromatic hydrocarbons (PAH) which have several potential adverse effects on environments and human health due to their toxicity, persistence, and carcinogenicity (Johnsen et al., 2005; Pumphrey and Madsen, 2007; Schafer et al., 2009). Microbes play an important role in the bioremediation of PAH-polluted sites. Microbial populations in polluted soils or sediments are very well studied (Gomes et al., 2005, 2007; Leys et al., 2005; Singleton et al., 2005; Ni Chadhain et al., 2006; Zhou et al., 2006; Flocco et al., 2009; Liang et al., 2009). Degradative populations were frequently affiliated to the genera *Sphingomonas*, *Polaromonas*, *Burkholderia*, *Pseudomonas*, *Mycobacterium*, *Nocardia*, and *Rhodococcus*. The type of PAH substrates (Singleton et al., 2005; Ni Chadhain et al., 2006; Gray et al., 2011) together with several biotic and abiotic factors, e.g., plant exudates (Sipila et al., 2008; Cebron et al., 2011) or particle sizes of soil minerals (Uyttebroek et al., 2006), were shown to influence the composition and abundance of total or degradative bacterial populations in polluted soils. However, the dynamics of microbial communities in unpolluted soils which are under the threat of PAH pollutions were only rarely explored.

Polycyclic aromatic hydrocarbons or their metabolites may have toxic effects on microbes (Eom et al., 2007). Previously unpolluted soils which were reported to have a low abundance of PAH-degrading populations (Johnsen et al., 2007; Flocco et al., 2009; Ding et al., 2010) and thus detoxification of PAH are assumed to be retarded. As a consequence, PAH pollution of non-adapted soils might reduce soil microbial diversity and cause more severe effects on soil microbial function as a high microbial diversity is assumed to be important for the proper functioning of soil ecosystems (Giovannoni, 2004; Bell et al., 2005). Toxic effects of PAH on plants (Li et al., 2011) and animals (Brown et al., 2004) were reported, but so far microbial ecology studies mainly focused on microbes which are able to degrade the pollutants or their metabolites. In a study by Sipila et al. (2008) the relative abundance of *Acidobacteria* subgroup GP1 was lower in the PAH-polluted bulk soil and rhizosphere than in the non-polluted soil.

In a previously described experiment, a Cambisol and a Luvisol soil contrasting in their texture were either spiked with phenanthrene or not and incubated in soil microcosms for 63 days (Ding et al., 2010). The diversity and abundance of enriched populations carrying PAH-hydroxylating dioxygenase (*PAH-RHD*α) genes depended on the soil type (Ding et al., 2010). Here we investigated for the same experiment how the bacterial communities change after spiking of the Cambisol and Luvisol with phenanthrene, in comparison to the unpolluted controls, by analyzing 16S rRNA gene fragments amplified from total community (TC) DNA by denaturing gradient gel electrophoresis (DGGE). An integrated data processing pipeline was developed for the analysis of data from pyrosequencing of 16S rRNA gene amplicons from the spiked soils and unpolluted controls after 63 days of incubation, in order to systematically identify taxa with increased or decreased abundance, to compare community structure and diversity of total and important groups between different samples.

## Materials and Methods

### Experimental design

Details of the experimental setup were described previously (Ding et al., 2010). Briefly, Cambisol and Luvisol soil samples taken from the long-term observation sites in Ultuna (Sweden) and Scheyern (Germany), respectively, were contaminated with phenanthrene to reach a final concentration of 2 mg g^−1^ soil. Four replicates of each soil, phenanthrene-spiked or not, were incubated at room temperature (23°C) in the dark. Samples were taken on days 0, 21, and 63 after phenanthrene spiking and kept at −20°C before DNA extraction with Bio-101 DNA spin kit for soil (QBiogene, Heidelberg, Germany).

### DGGE analysis of 16S rRNA gene fragments

Bacterial 16S rRNA gene fragments from the soil samples were amplified with the primers F984-GC and R1378 as described by Heuer et al. (1997). A semi-nested or nested PCR approach was applied for amplification of 16S rRNA genes of *Actinobacteria*, *Alphaproteobacteria*, *Betaproteobacteria*, or *Pseudomonas* as previously described (Heuer et al., 1997; Gomes et al., 2001; Costa et al., 2007). All primers used in this study are provided in Table A1 in Appendix. DGGE of the 16S rRNA gene amplicons was performed according to Gomes et al. (2005), using a double gradient gel composed of 46.5–65% denaturants (100% denaturant was defined as 7 M urea and 40% formamide and 6.2–9% of mixture of bis-acrylamide and acrylamide (1:37.5). The following electrophoresis was performed at a constant voltage of 140 V for 17 h at 58°C in 1× Tris-acetate-EDTA buffer with a PhorU2 apparatus (Ingeny, Goes, Netherlands). The DGGE gels were silver stained according to Heuer et al. (2001). GelCompar II 4.5 was used for pairwise comparisons of microbial DGGE profiles. Dendrograms were constructed based on pairwise Pearson correlation indices by means of unweighted pair group method using arithmetic averages (UPGMA). The pairwise Pearson correlation indices were used to test for significant treatment effects by application of the previously described PERMTEST software (Kropf et al., 2004). Cloning and sequencing of the selected bands from DGGE gels were done as previously described (Gomes et al., 2008). The partial 16S rRNA gene sequences excluding the primers were classified by the Naïve Bayesian rRNA Classifier of the Ribosomal Database Project^1^ and BLASTN in the GenBank database.^2^

### Pyrosequencing

Three replicate samples per treatment and control from day 63 were studied more in depth by barcoded pyrosequencing. PCR amplification and sequencing were done at Biotechnology Innovation Center (BIOCANT, Cantanhede, Portugal). Briefly, the hypervariable V3–V4 regions of 16S rRNA genes were amplified with bacterial primers 338F and 802R (RDP’s Pyrosequencing Pipeline^3^) which were fused to the 454 A and B adaptors, respectively. Standard PCR reaction conditions were employed for reactions with Fast Start polymerase (Roche, Pensberg, Germany), 3 mM MgCl_2_, 6% DMSO, 200 nM each primer and 200 mM dNTP. The PCR conditions were 94°C for 3 min, followed by 35 cycles of 94°C for 30 s, 44°C for 45 s, and 72°C for 60 s, and a final elongation step at 72°C for 2 min. Sequencing was performed on a 454 Genome Sequencer FLX platform according to standard 454 protocols (Roche – 454 Life Sciences, Branford, CT, USA).

### Sequence and statistical analyses

A semi-automatic pipeline for analyzing 16S rRNA gene sequences was integrated by Perl^4^ (5.12). The pipeline consists of two major parts: an operational taxonomic unit (OTU) report generator and an OTU report analyzer (supplement).

Denoizing, multiple alignments, OTU assignment, classification, and generation of OTU reports were linked according to the following steps and criteria: the unpaired region for each sequence was truncated based on standalone BLASTN analysis against a SILVA 16S rRNA gene database (Pruesse et al., 2007). Only sequences of more than 200 bp were analyzed. The software package Mothur (v1.14.0; Schloss et al., 2009; Schloss, 2010) was used for multiple alignments and OTU assignment. Classification of sequences was done by using the Naïve Bayesian classifier at >80% confidence (Wang et al., 2007). Aligned sequences, their corresponding taxonomy, as well as OTU assignment were stored in a local MYSQL database. A Perl script including the package database interface (DBI) was used to construct an OTU level report with each row representing one OTU containing taxonomic position (domain, phylum, class, order, family, and genus) and number of sequences for each sample.

Analyses based on the OTU (>97%) report were done with R^5^ (2.12). To compare the community structure between samples, cluster analyses were performed, including all taxonomic groups with more than 50 OTUs, based on the pairwise Pearson correlation which is suitable to compare samples with a different number of sequence reads. The reliability of clusters was tested by 500 times bootstrap analyses. The difference in community structure between treatments was measured using the average of Pearson correlation within treatment minus average of Pearson correlation between treatments. Rarefaction analysis for different treatments and different taxonomic groups was also performed with R to compare the detected diversity between treatments. Diversity indices such as Chao1, Pielous’s evenness, and Shannon were also analyzed in a similar manner as the rarefaction analysis to alleviate the bias caused by the different numbers of sequence reads per sample.

Discriminative taxa between treatments or soil types were identified by Tukey’s honest significance tests under a generalized linear model via a logistic function for binomial data with the package multcomp (Hothorn et al., 2008). The analyses were performed systematically from phylum to species level (OTU > 97% similarity). Representative sequences for those discriminative OTUs (>97% sequences similarity) as well as their closest related 16S rRNA gene sequences were aligned with Mothur. A neighbor-joining phylogenetic tree was constructed and tested using software package SeaView4 (Gouy et al., 2010). Compression of the branches was performed to reduce the complexity of the phylogenetic tree generated. Nodes in the phylogenetic tree were marked for compression at defined distances using a local Perl package (gardener) including TreeIO (Bioperl) and DBI. Phylogenetic trees were edited with software Archaeopteryx (Han and Zmasek, 2009).

## Results

### Responses of soil bacterial communities to phenanthrene depending on soil type, exposure time, and taxonomic group as revealed by PCR-DGGE

Community fingerprints of *Bacteria*, *Pseudomonas*, *Actinobacteria*, *Beta-*, and *Alphaproteobacteria* were clustered by UPGMA based on Pearson correlation indices. Phenanthrene significantly affected the bacterial community structure in both soils (Table 1).

**Table 1:** **Percent dissimilarity between microbial DGGE fingerprints of different taxa from phenanthrene-spiked and control soils**.

Days after spiking	Soil type	*Bacteria*	*Alphaproteobacteria*	*Betaproteobacteria*	*Actinobacteria*	*Pseudomonas*	*Fungi*
21	Luvisol	46*	25	25*	5	4	0
	Cambisol	17*	8	12*	2	23*	2
63	Luvisol	63*	38*	60*	41*	7*	2
	Cambisol	36*	56*	46*	30*	8	11*

*Significant (*p* ≤ 0.05) difference between phenanthrene-spiked and control soils. Percent dissimilarity = average within-group pairwise Pearson’s correlation − average between-group pairwise Pearson’s correlation.

**Figure A1:**
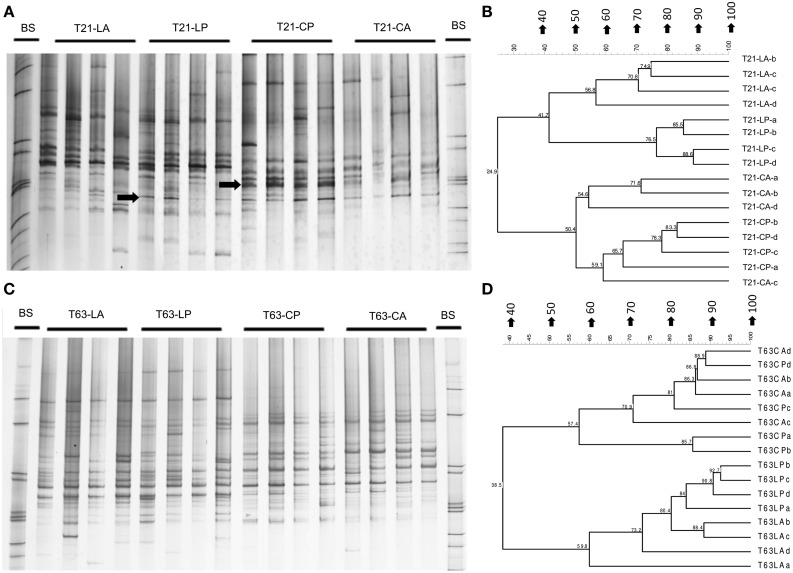
***Pseudomonas*-specific *gacA* DGGE fingerprint (A,C) and the corresponding UPGMA clusters (B,D) using pairwise Pearson correlation indices for soil spiked with phenanthrene (LP: phenanthrene-spiked Luvisol, CP: phenanthrene-spiked Cambisol) or not (LA: Luvisol control, CA: Cambisol control) on days 21 and 63**. Arrows showing bands with enhanced intensities in phenanthrene spiked soils. BS: bacterial standards.

**Figure A2:**
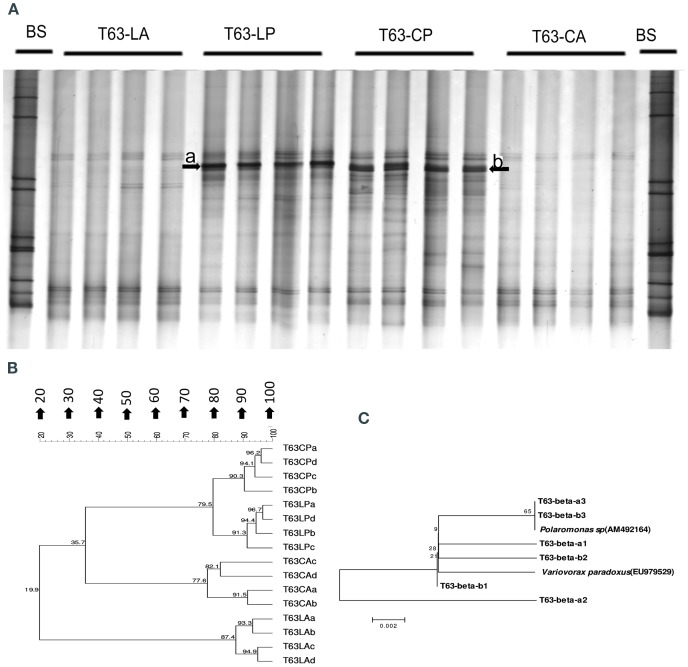
***Betaproteobacteria*-specific DGGE fingerprint (A), corresponding UPGMA clusters (B) for soils spiked with phenanthrene (LP: phenanthrene-spiked Luvisol, CP: phenanthrene-spiked Cambisol) or not (LA: Luvisol control, CA: Cambisol control) and neighbor-joining rooted phylogenetic tree (C) based on sequences (three clones were sequenced for each band) behind dominant enriched bands (band a and band b) in both polluted soils on day 63 (T63)**.

**Figure A3:**
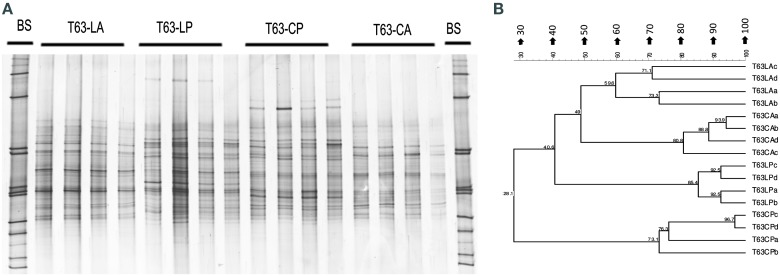
***Alphaproteobacteria*-specific DGGE fingerprints (A) and the corresponding UPGMA clusters (B) for soil spiked with phenanthrene (LP: phenanthrene-spiked Luvisol, CP: phenanthrene-spiked Cambisol) or not (LA: Luvisol control, CA: Cambisol control) on day 63 (T63)**.

**Figure A4:**
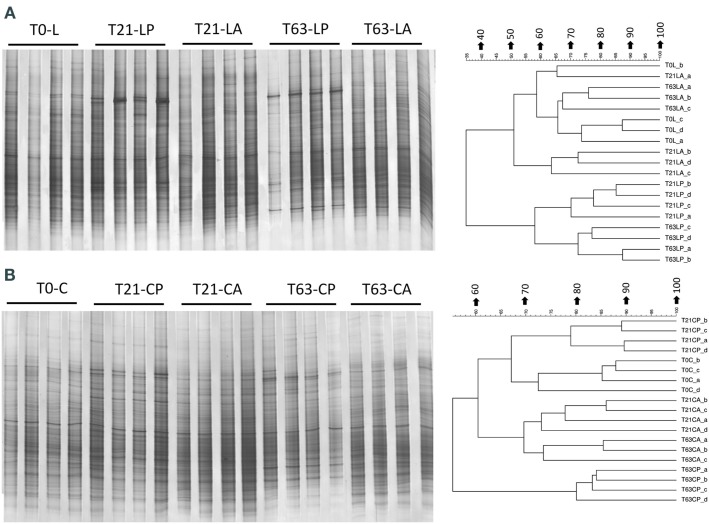
**Bacterial DGGE fingerprint (A,C) and the corresponding UPGMA clusters (B,D) for soil spiked with phenanthrene (LP: phenanthrene-spiked Luvisol, CP: phenanthrene-spiked Cambisol) or not (LA: Luvisol control, CA: Cambisol control) on day 21 (T21) and 63 (T63) and the initial soils (L: Luvisol and C: Cambisol) on day 0 (T0)**.

The bacterial taxonomic groups analyzed responded differently to phenanthrene spiking (Table 1). Despite the relatively high variability of the *Pseudomonas*-specific *gacA* fingerprints some differentiating bands were detected in the patterns of phenanthrene-polluted Luvisol and Cambisol on day 21 (Figures A1A,B in Appendix). A pronounced difference (*d* = 23%) of the *Pseudomonas* community structure between phenanthrene-spiked Cambisol and the control was observed (Table 1). But on day 63, no differentiating bands were identified in response to the phenanthrene contamination in the *Pseudomonas* fingerprints (Figures A1C,D in Appendix), and the dissimilarity of *Pseudomonas* microbial DGGE fingerprints between phenanthrene-polluted soil and the corresponding control was small (Table 1). In contrast to *Pseudomonas* communities, a difference of the actinobacterial communities of phenanthrene-polluted and control soils was observed only on day 63 but not on day 21 (Table 1). Responses of betaproteobacterial communities to phenanthrene spiking were observed for both soils on days 21 and 63. The populations (accession numbers: JF810414-JF810419) behind the bands with strongly increased intensity (most dominant responder) in both soils shared the highest sequence similarity with *Polaromonas* sp. (AM492164; Figures A2A–C in Appendix). The alphaproteobacterial community patterns for soil samples taken on day 21 displayed a high variability among replicates. But some differentiating bands were identified when comparing polluted and unpolluted Luvisol (data not shown). On day 63, significant differences were observed between polluted and non-polluted treatments for both soils (Table 1 and Figures A3A,B in Appendix). As for the total bacterial community, differences of the alphaproteobacterial, betaproteobacterial, and actinobacterial community between the polluted soils and the corresponding controls strongly increased with time in both soils, while the community of *Pseudomonas* became more similar between phenanthrene-treated and non-treated soils (Table 1). Changes in the bacterial community structure of the untreated control soils during the experiment were minor for Cambisol and not detectable for Luvisol, indicating that microbial community changes were directly or indirectly induced by the phenanthrene spiking (Figure A4 in Appendix). Based on the *d*-values (Table 1), differences in the bacterial community structure after phenanthrene spiking were more pronounced in Luvisol than in Cambisol at both samplings.

### Soil type and taxonomic group dependent effects of phenanthrene spiking on bacterial community structure and diversity as revealed by 16S rRNA gene amplicon sequencing

**Figure A5:**
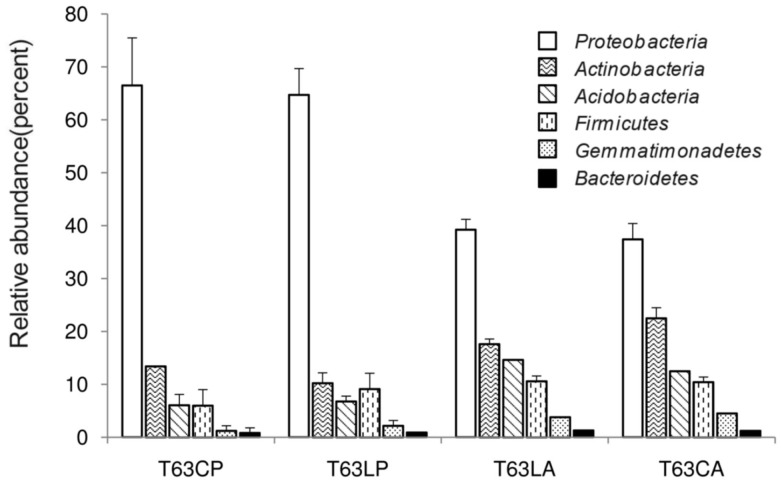
**Relative abundance of dominant phyla detected for soils spiked with phenanthrene (LP: phenanthrene-spiked Luvisol, CP: phenanthrene-spiked Cambisol) or not (LA: Luvisol control, CA: Cambisol control) on day 63 (T63)**.

Three replicate samples per treatment from day 63 were analyzed by barcoded pyrosequencing of 16S rRNA genes. Altogether 34,054 sequences for all 12 samples were examined, of which 31,201 sequences could be classified to 21 phyla. The remaining 2,853 sequences were only classified as *Bacteria*. More than 40% could not be reliably classified at genus level indicating novel diversity not yet described (Table A2 in Appendix). The most abundant phyla in both soils belonged to the *Proteobacteria*, *Actinobacteria*, *Acidobacteria*, *Firmicutes*, and *Gemmatimonadetes* (Figure A5 in Appendix).

**Figure 1:**
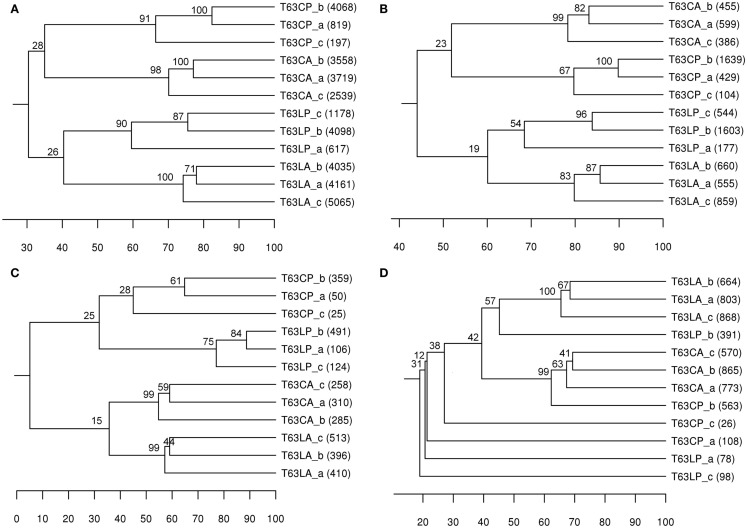
**UPGMA cluster analysis of bacterial communities’ structure based on Pearson correlation using OTUs (> 97% sequence similarity) as the species data**. Number in brackets: number of sequences obtained for the sample. Numbers on node: Bootstrapping value for each node means the percent frequency of all samples under the node grouping exclusively together. **(A)** total bacterial community; **(B)** *Alphaproteobacteria*; **(C)** *Betaproteobacteria*; **(D)** *Actinobacteria*. T63, 63 days after phenanthrene spiking; CA: Cambisol control, CP: phenanthrene-spiked Cambisol, LA: Luvisol control, LP: phenanthrene-spiked Luvisol.

A taxonomic report containing 8,889 OTUs at species level (>97% sequences similarity) was obtained for comparing community structures. Phenanthrene spiking strongly influenced the total bacterial community structure in both soils. The differences between polluted soils and their corresponding control were comparable for both soils (37% for Cambisol and 30% for Luvisol; Figure 1A). Bootstrap analysis confirmed the high reliability of the formed clusters for each treatment (bootstrap value > 90), suggesting that the total bacterial community differed between treated and control soils. The difference (*d* = 30%) between two polluted soils was similar to the difference (*d* = 34%) between the control soils (Figure 1A), indicating that bacterial communities in both soils did not converge or diverge after pollution. To compare DGGE and pyrosequencing in analyzing the community structure of bacterial taxonomic groups, *Alphaproteobacteria*, *Betaproteobacteria*, and *Actinobacteria* were also analyzed using OTUs belonging to these taxa, respectively. Like DGGE, clear soil type and treatment clusters were formed for alphaproteobacterial communities (Figure 1B). In contrast to the DGGE results the differences between polluted soils and the corresponding controls were comparable for both soil types (Figure 1B). Pyrosequencing analysis additionally showed that the topology of the UPGMA clusters for *Alphaproteobacteria* was highly similar to that observed for the total bacterial community, suggesting that the dominant members of the bacterial communities and responders belonged to *Alphaproteobacteria*. Both DGGE and pyrosequencing analyses suggested that differences between betaproteobacterial communities of polluted and unpolluted Luvisol (*d* = 66%) were stronger than for Cambisol (*d* = 43%; Table 2 and Figure 1C). The polluted soils shared more similarity to each other than to their corresponding control soil. Additionally, pyrosequencing analysis showed that replicates were more similar for the polluted Luvisol (>74%) than the control (55–59%), indicating convergence of the betaproteobacterial communities in the Luvisol after phenanthrene pollution. DGGE analysis revealed a significant effect of the phenanthrene spiking on actinobacterial communities in both soils. The similarities shared by replicates within each type of polluted soil were high. Pyrosequencing analysis confirmed the significant difference between polluted soils and the corresponding control except for one replicate of the control Cambisol (Figure 1D). Unlike the DGGE analysis, the similarity among replicates of dominant actinobacterial communities in both polluted soils was low, probably due to the high diversity of *Actinobacteria* and the rather limited amount of sequences acquired for some of the phenanthrene-spiked soils.

**Table 2:** **Taxa enriched in the polluted soils or with significant (*p* < 0.05) different relative abundance (%) between two soils**.

Phylum	Class	Order	Family	Genus	T63CA (3272 ± 640)^a^	T63CP (1695 ± 2079)	T63LA (4420 ± 562)	T63LP (1964 ± 1869)
***Proteobacteria**^b^*	***Alphaproteobacteria***	***Sphingomonadales***	***Sphingomonadaceae***	***Novosphingobium***	0 ± 0	1 ± 0***	**0.3 ± 0^c^**	0.8 ± 0***
				***Sphingomonas***	3.4 ± 1	23.4 ± 4***	**4 ± 1**	18 ± 5***
				*Sphingosinicella*	0 ± 0	0.1 ± 0	0 ± 0	0.1 ± 0*
		***Caulobacterales***	***Caulobacteraceae***	*Caulobacter*	0.07 ± 0	0.1 ± 0*	0 ± 0	0.1 ± 0
				***Phenylobacterium***	0.3 ± 0	1.2 ± 1***	0.3 ± 0	0.9 ± 0***
		*Rhizobiales*	*Bradyrhizobiaceae*	*Bosea*	0 ± 0	0.2 ± 0***	0 ± 0	0.1 ± 0
				***Afipia***	0 ± 0	0.2 ± 0**	0 ± 0	0.4 ± 0***
	*Betaproteobacteria*	***Burkholderiales***	***Comamonadaceae***	***Polaromonas***	**0.2 ± 0**	4.1 ± 2***	0 ± 0	6.7 ± 3***
			***Burkholderiaceae***	***Burkholderia***	0 ± 0	0.1 ± 0*	0 ± 0	0.2 ± 0*
			*Oxalobacteraceae*	*Duganella*	0 ± 0	0.1 ± 0*	0.1 ± 0	0.2 ± 0
				*Herbaspirillum*	0 ± 0	0 ± 0	0 ± 0	0.4 ± 0***
				***Rhodanobacter***	0 ± 0	0.3 ± 0***	0 ± 0	1.1 ± 0 ***
	*Gammaproteobacteria*	*Xanthomonadales*	*Xanthomonadaceae*	*Dokdonella*	0.6 ± 0	0.5 ± 0	0.6 ± 0	1.8 ± 1***
				*Dyella*	0 ± 0	0 ± 0	0.1 ± 0	0.2 ± 0*
				*Pseudoxanthomonas*	0 ± 0	0.4 ± 1*	0 ± 0	0 ± 0
		*Pseudomonadales*	*Pseudomonadaceae*	*Pseudomonas*	0.1 ± 0	0.6 ± 1***	0.1 ± 0	0.3 ± 0
		***Legionellales***	*Coxiellaceae*	*Aquicella*	0.1 ± 0	0.3 ± 0	0.1 ± 0	0.3 ± 0***
			*Legionellaceae*	*Legionella*	0 ± 0	0 ± 0	0 ± 0	0.3 ± 0***
	*Deltaproteobacteria*	***Bdellovibrionales***	***Bacteriovoracaceae***	***Peredibacter***	0.1 ± 0	0.3 ± 1***	0 ± 0	0.2 ± 0***
		*Nannocystineae*	*Nannocystaceae*	*Nannocystis*	0 ± 0	0 ± 0	0 ± 0	0.1 ± 0**
		*Sorangiineae*	*Polyangiaceae*	*Byssovorax*	0.1 ± 0	0 ± 0	0.1 ± 0	1 ± 1***
		*Desulfuromonadales*			0.1 ± 0	0 ± 0	**0.6 ± 0**	0.5 ± 0
*Actinobacteria*	*Actinobacteridae*	***Corynebacterineae***	***Mycobacteriaceae***	***Mycobacterium***	0.1 ± 0	0.2 ± 0**	0.1 ± 0	0.5 ± 0**
		*Micromonosporineae*	*Micromonosporaceae*	*Dactylosporangium*	0 ± 0	0 ± 0	0 ± 0	0.2 ± 0***
		*Frankineae*			**1.1 ± 0**	0.4 ± 0	0.7 ± 0	0.1 ± 0
		*Micrococcineae*			**3 ± 1**	2.9 ± 1	1.9 ± 0	1.2 ± 1
		*Streptomycineae*			**1.2 ± 0**	0.7 ± 0	0.8 ± 0	0.3 ± 0
	*Acidimicrobidae*				1.3 ± 0	0.9 ± 0	**1.9 ± 0**	0.5 ± 0
	*Rubrobacteridae*				**7.8 ± 1**	3.3 ± 1	4.4 ± 1	2.3 ± 1
*Bacteroidetes*	*Sphingobacteria*	*Sphingobacteriales*	*Sphingobacteriaceae*	***Mucilaginibacter***	0 ± 0	0.2 ± 0***	0 ± 0	0.3 ± 0***
*Acidobacteria*					12.5 ± 0	6.1 ± 2	**14.6 ± 0**	6.8 ± 1
*Gemmatimonadetes*	*Gemmatimonadetes*	*Gemmatimonadales*	*Gemmatimonadaceae*	*Gemmatimonas*	**4.5 ± 0**	1.2 ± 1	3.8 ± 0	2.2 ± 1

^a^Average of sequence number ± standard deviation; significant higher relative abundance in the polluted soils (*: 0.01 < *p* < 0.05; **: 0.001 < *p* < 0.01; ***: *p* < 0.001). ^b^Bold text, taxa of significant high relative abundance in both polluted soils. ^c^Bold numbers, significant higher relative abundance in the Luvisol or Cambisol. Relative abundance (%): 100 × (sequences belonging to the taxa)/total sequences detected for samples. T63, 63 days after phenanthrene spiking; CA: Cambisol control, CP: phenanthrene-spiked Cambisol, LA: Luvisol control, LP: phenanthrene-spiked Luvisol.

**Figure 2:**
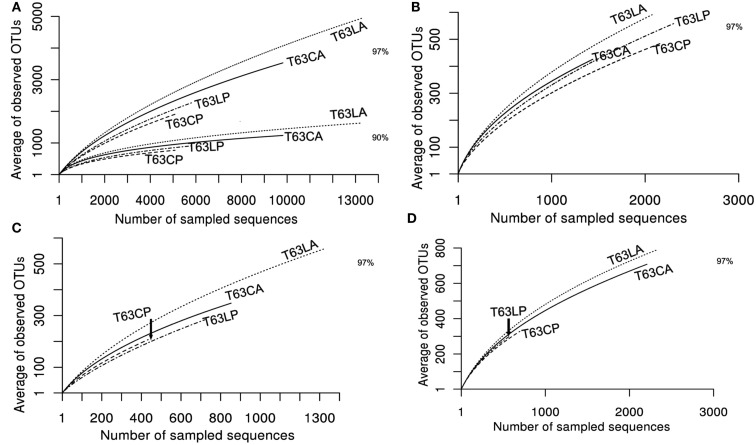
**Plot of rarefaction curves for soils spiked with phenanthrene (LP, CP) or not (LA, CA) on day 63**. **(A)** Total bacteria; **(B)** *Alphaproteobacteria*; **(C)** *Betaproteobacteria*; **(D)** *Actinobacteria*. T63, 63 days after phenanthrene spiking; CA: Cambisol control, CP: phenanthrene-spiked Cambisol, LA: Luvisol control, LP: phenanthrene-spiked Luvisol.

**Figure A6:**
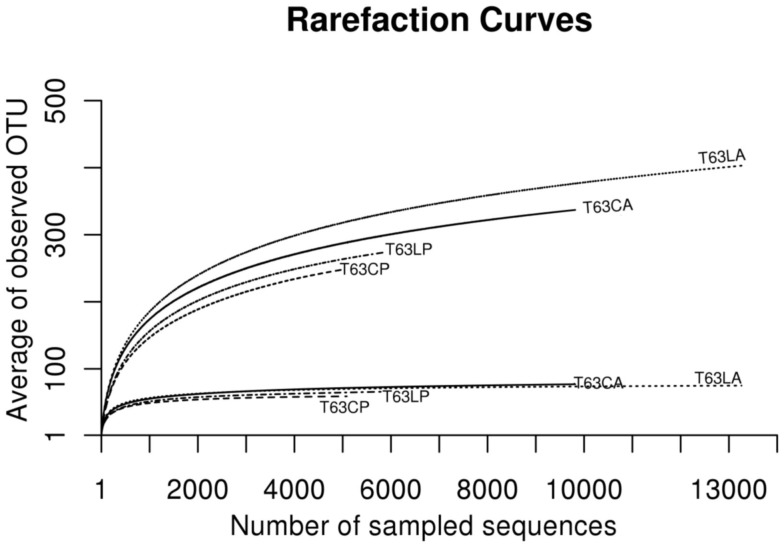
**Plot of rarefaction curves at 80 and 70% similarities for soils spiked with phenanthrene (LP: phenanthrene-spiked Luvisol, CP: phenanthrene-spiked Cambisol) or not (LA: Luvisol control, CA: Cambisol control) on day 63 (T63)**.

**Figure A7:**
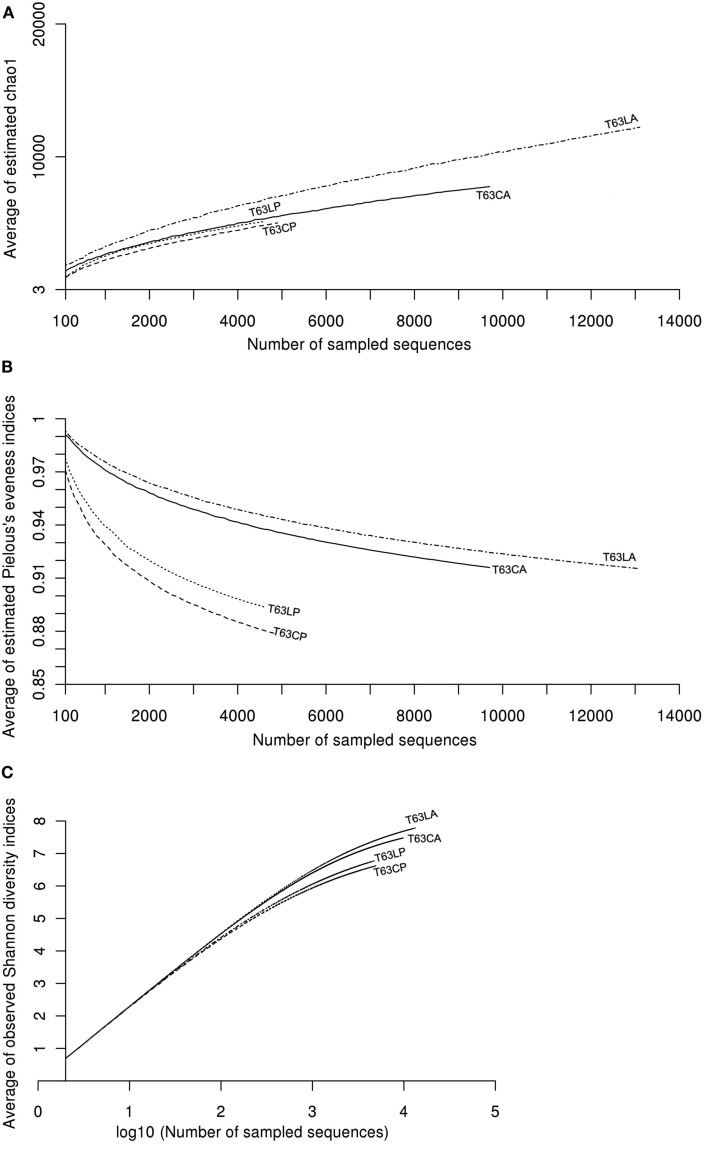
**Plot of curves of average diversity indices [(A): CHAO1, (B): Pielous’s evenness, and (C): Shannon] for soils spiked with phenanthrene (LP: phenanthrene-spiked Luvisol, CP: phenanthrene-spiked Cambisol) or not (LA: Luvisol control, CA: Cambisol control) on day 63 (T63)**. Average diversity indices were calculated in a similar way to rarefaction analysis with 100 times repeating sampling.

To compare the diversity of detected sequences from different treatments, rarefaction analyses were performed based on OTUs defined at four similarity levels (97% and 90% = Figure 2A, 80% and 70% = Figure A6 in Appendix). The bacterial diversity was lower in the polluted soils than in the unpolluted control at all similarity levels (Figure 2A and Table A3 in Appendix). The lowest diversity was found for the polluted Cambisol (Figure 2A). A lower bacterial diversity (richness and evenness) in the polluted soils was also confirmed by Chao1 (Figure A7A in Appendix), Pielous’s evenness (Figure A7B in Appendix), and Shannon index (Figure A7C in Appendix). For *Alphaproteobacteria* (Figure 2B), *Betaproteobacteria* (Figure 2C), and *Actinobacteria* (Figure 2D), the detected diversities were also lower for polluted soils than for the controls. The average of observed OTUs for 400 sequences was used to compare the diversity in phenanthrene-spiked and control soils (*Alphaproteobacteria*, *Betaproteobacteria*, and *Actinobacteria* could be analyzed with this amount of sequences). The percentage of reduction in diversity after pollution depended on the taxonomic group and the soil type. For the polluted Luvisol, the highest decrease in diversity was observed for *Betaproteobacteria* (27%), followed by *Bacteria* (15%), *Alphaproteobacteria* (13%), and *Actinobacteria* (9%). For the polluted Cambisol, the decrease in betaproteobacterial diversity was only 9%, while a similar decrease in diversity was found for *Bacteria*, *Alphaproteobacteria*, and *Actinobacteria* in both polluted soils.

### Numerous common and soil type specific taxa enriched in phenanthrene-polluted soils

To identify those taxa which were enriched in the polluted soils, multiple Tukey’s tests were applied (unadjusted *p* < 0.05). Phenanthrene spiking selectively enriched diverse bacterial taxonomic groups (Table 2). About 10% of the genera detected were significantly enriched in one or both polluted soils. Most genera belonged to *Proteobacteria* (23 genera), and few to *Actinobacteria* (2 genera) or *Bacteroidetes* (1 genus; Table 2). Many genera were never reported being involved in PAH degradation, e.g., *Sphingosinicella*, *Afipia*, *Duganella*, *Herbaspirillum*, *Rhodanobacter*, *Dokdonella*, *Dyella*, *Aquicella*, *Peredibacter*, *Legionella*, *Nannocystis*, *Byssovorax*, *Dactylosporangium*, and *Mucilaginibacter*. The relative abundance of most enriched genera was still low (<0.4%) in both polluted soils. *Sphingomonas*, *Polaromonas*, *Novosphingobium*, *Phenylobacterium*, *Burkholderia*, *Mycobacterium*, and *Mucilaginibacter* were enriched in both polluted soils. Among them, *Sphingomonas* (>18%) and *Polaromonas* (>4%) became the most dominant genera in both soils. In contrast to these genera commonly enriched in both polluted soils, *Herbaspirillum*, *Dokdonella*, *Dyella*, *Legionella*, and *Dactylosporangium* were enriched in the polluted Luvisol, while *Pseudoxanthomonas* showed an increased abundance only in the polluted Cambisol. Interestingly, the genus *Dokdonella* had a similar relative abundance (0.6%) in both control soils, but an increased abundance of this genus was only observed in the polluted Luvisol (Table 2). Further analysis at the species level confirmed that the most abundant *Dokdonella* OTUs were present in both control soils but a significantly increased abundance was observed only in the polluted Luvisol. Other examples of soil type specific responses were observed for *Novosphingobium*, *Sphingomonas*, and *Polaromonas* (Table 2).

To pinpoint those species which significantly differed between phenanthrene-polluted and non-polluted soils, all OTUs at the species level were subjected to Tukey’s tests. A total of 88 OTUs were identified as significantly enriched in the polluted soils, and the majority of them were affiliated to *Proteobacteria* and *Actinobacteria*. The diversity of enriched OTUs was higher in the polluted Luvisol (69 OTUs belonging to *Proteobacteria* or *Actinobacteria*) than in the polluted Cambisol (49 OTUs belonging to *Proteobacteria*, *Actinobacteria*, *Acidobacteria*, or *Bacteroidetes*) at species level. Interestingly, 30 out of 88 OTUs (Table A4 in Appendix) were commonly enriched in both polluted soils. Some enriched OTUs (*Sphingomonadaceae*) were also abundant in the control soils. Eight out of the 10 most abundant and significantly enriched OTUs were more frequently detected than average in the control soils. Three of them even ranked among the most detected seven OTU in the control Luvisol. In contrast, the most dominant OTUs detected for both polluted soils were not detected from their corresponding control at all. Their relative abundance was extremely high, reaching 3.9% in the polluted Cambisol and 2.5% in the polluted Luvisol. Interestingly, they were affiliated to *Sphingomonas* (*Sphingomonadaceae*) and *Polaromonas* (*Burkholderiales*), respectively, corresponding to the results in the previous study on *PAH-RHD*α genes.

### Diverse soil type specific taxa with decreased abundance in the polluted soils

To identify those taxa numerically reduced (termed in the following as diminished OTUs) in the polluted soils, stringent criteria were applied. Only those OTUs were regarded as diminished OTUs which were five times more often detected than average in the control soil but were never detected in the corresponding polluted soils, or which were at least four times more often detected than average and significantly higher (*p* < 0.05) in relative abundance in control soil than in the corresponding polluted soil.

**Figure 3:**
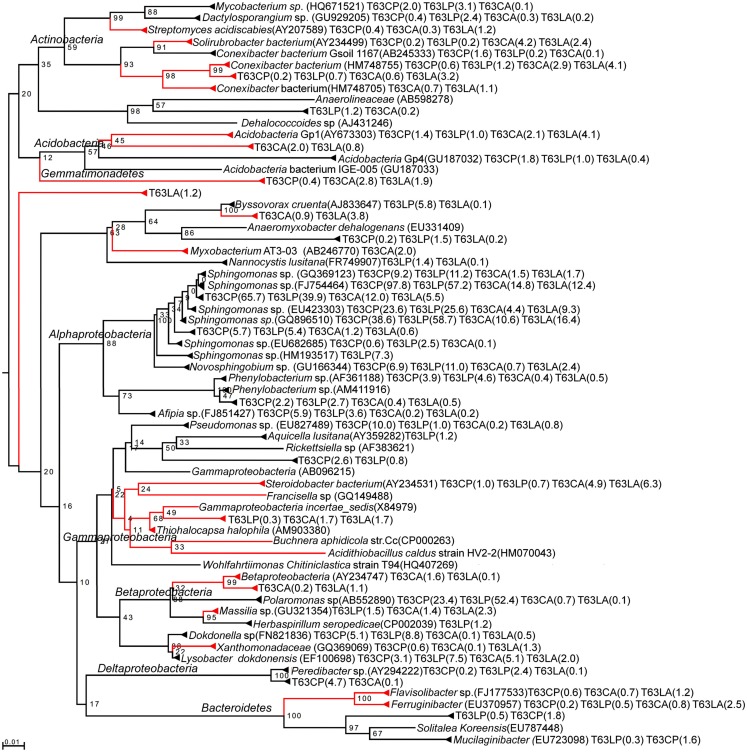
**Neighbor-joining rooted phylogenetic tree based on multiple alignments of representative sequences for discriminative OTUs**. Red branch: OTUs numerically reduced in the polluted soils. Black branch: OTUs enriched in the polluted soils. Values at each node = (bootstrap value/100) × 100. Number in brackets: relative abundance in %. T63, 63 days after phenanthrene spiking; CA: Cambisol control, CP: phenanthrene-spiked Cambisol, LA: Luvisol control, LP: phenanthrene-spiked Luvisol.

A total of 30 OTUs were identified, belonging to five phyla (*Proteobacteria*, *Actinobacteria*, *Bacteroidetes*, *Acidobacteria*, and *Gemmatimonadetes*). Compared to those enriched OTUs, the OTUs with decreased abundance seemed more soil type specific. Only one OTU affiliated to *Massilia* sp. (D84590) was found to be reduced in both polluted soils. Most diminished OTUs belonged to the *Proteobacteria*, but no one was affiliated with *Alphaproteobacteria* to which most enriched OTUs belonged. The diminished OTUs belonging to the *Proteobacteria* were affiliated to *Gammaproteobacteria* (eight OTUs), *Deltaproteobacteria* (four OTUs), or *Betaproteobacteria* (four OTUs). To compare the diminished OTUs and enriched OTUs phylogenetically, representative sequences for each OTU as well as their closest related reference sequences were retrieved from the SILVA 16S rRNA gene database (Pruesse et al., 2007) and subjected to phylogenetic analysis (Figure 3). Interestingly, the diminished OTUs and those enriched OTUs belong to different phylogenetic branches (Figure 3). For example, diminished OTUs belonging to *Actinobacteria* were affiliated with *Conexibacter*, *Solirubrobacter*, and *Streptomyces* while sequences of enriched OTUs were affiliated to *Mycobacterium* and *Dactylosporangium* (Figure 3). Other examples were also observed for *Acidobacteria* and *Bacteroidetes* (Figure 3).

## Discussion

The response to phenanthrene spiking of bacterial communities in two previously unpolluted soils was explored by DGGE and pyrosequencing analysis of 16S rRNA gene fragments amplified from TC DNA. A novel semi-automatic pipeline for analyzing 16S rRNA gene sequences consisting of an OTU report generator and OTU report analyzer allowed us to determine the community structure but also to reveal taxonomic affiliation of the responders to phenanthrene spiking. The deviation of the bacterial communities between the spiked and non-spiked control increased over time, suggesting that specific bacteria grew on the added substrate or its metabolites. The concentration of phenanthrene in both soils continually decreased after spiking (Ding et al., 2010). Many enriched ribotypes belonged to genera that were never reported as responders to PAH pollution. However, the bacterial populations which showed the strongest enrichment in both soils belonged to genera such as *Sphingomonas*, *Polaromonas*, which were previously found to be involved in PAH degradation in polluted soils or sediments (Jeon et al., 2003; Singleton et al., 2006; Sun et al., 2010; Jones et al., 2011). Several bacterial isolates affiliated to the two genera were shown to grow on media with one of PAH substrates as sole carbon source (Jeon et al., 2006; Kobayashi et al., 2009; Schuler et al., 2009), suggesting they could be the dominant populations utilizing added PAH substrates. However, microorganisms were reported degrading organic pollutants cooperatively (Sorensen et al., 2002; Dejonghe et al., 2003; Guo et al., 2010) and the populations controlling the flux of metabolic intermediates were assumed to be important for accelerating bioremediation of polluted environments (Head et al., 2006).

In the present study, only a small proportion of enriched *Sphingomonas* populations seemed to carry *RHD*α-like genes (Ding et al., 2010) that are essential for the first step of the aerobic phenanthrene degradation, and that the majority was benefiting from the initial degraders by syntrophic interactions. This finding is supported by the GeoChip analysis (Ding et al., 2012) as most enriched organic pollutant remediation genes were linked with the degradation of one-ring aromatic hydrocarbons and chloro-hydrocarbons. Some of the enriched OTUs belonging to *Sphingomonas* were also highly abundant in the control soils from which no *PAH-RHD*α gene amplicons were previously detected (Ding et al., 2010), suggesting that dominant *Sphingomonas* probably had a better chance to utilize metabolic intermediates of phenanthrene.

Bacterial consortia consisting of functional complementary groups might facilitate synergistic degradation of organic pollutant by decreasing the possible inhibition effects of metabolic intermediates (Keck et al., 1989). A fast degradation of PAH substrates by co-culture of *Sphingomonas* and *Mycobacterium* was indeed observed by Guo et al. (2010).

Enrichment of *Polaromonas* (*Burkholderiales*) in both phenanthrene-spiked soils was evident by both DGGE and pyrosequencing analysis. *Polaromonas* is an important genus responsible for aromatic hydrocarbon degradation in soil or sediments (Jeon et al., 2003; Sun et al., 2010). *NagA* genes encode the key enzyme for naphthalene (PAH) degradation in *Polaromonas naphthalenivorans* CJ2 (Jeon et al., 2006). However, the enriched *Polaromonas* populations probably differed between polluted soils in this experiment, as *phnAc* was the dominant degradative gene in phenanthrene-spiked Luvisol but was not detected in the polluted Cambisol and both control soils (Ding et al., 2010). The gene *phnAc* was reported in strains of the genus *Burkholderia* (Laurie and Lloyd-Jones, 1999; Vacca et al., 2005), *Acidovorax* (Singleton et al., 2009), and *Delftia* (Hickey et al., 2012). All host bacteria of the *phnAc* gene reported so far belong to the order *Burkholderiales* (*Betaproteobacteria*). Based on the complete genome analysis of *P. naphthalenivorans* CJ2, Yagi et al. (2009) suggested that extensive horizontal gene transfer contributed to the evolution and metabolic versatility of this strain. Pyrosequencing analysis revealed that the most dominant OTU (2.5%) for phenanthrene-spiked Luvisol was also affiliated to *Polaromonas*, while this OTU was not detected for both control soils and was less abundant (0.3%) in polluted Cambisol. The relative abundance of this OTU (0.3%) was much lower than all OTUs affiliated to *Polaromonas* in the polluted Cambisol (4.1%). These results support that the majority of enriched *Polaromonas* populations in the polluted Cambisol likely also benefit from the initial degraders. The study of Yagi et al. (2009) also predicted that *P. naphthalenivorans* CJ2 can utilize aromatic hydrocarbons such as benzoate and biphenyl which share metabolic pathways with intermediates in the degradation of phenanthrene or other PAH.

In the present study, pyrosequencing data revealed that the relative abundance of *Pseudomonas* was significantly higher in the polluted Cambisol than in the corresponding control (Table 2), suggesting that some *Pseudomonas* bacteria grew in the polluted Cambisol. *Pseudomonas* was reported to be enriched in PAH-polluted sites (Edlund and Jansson, 2008; Jones et al., 2011). Unlike the pyrosequencing data, the DGGE analysis did not indicate an effect of phenanthrene on *Pseudomonas* which may be explained by the inefficient amplification of some *Pseudomonas* populations by the *gacA* primers (Costa et al., 2007). The role of the enriched *Pseudomonas* in the polluted Cambisol remains to be elucidated as no *nahAc*-like genes were found in the previous study on *PAH-RHD*α genes (Ding et al., 2010). *NahAc*-like genes are often located on IncP-9 plasmids (Dennis and Zylstra, 2004; Sota et al., 2006; Heinaru et al., 2009). The host range of IncP-9 plasmids is confined mainly to *Pseudomonas*. In our experiment, *oriV-rep* regions of IncP-9 plasmids were detected for most soil samples but no enhanced abundance of IncP-9 plasmids was found for the polluted soils (data not shown).

Pyrosequencing analysis revealed that the bacterial diversity [richness (Chao1, rarefaction analysis) and evenness (Pielous’s evenness indices)] was lower in the phenanthrene-spiked soils than the non-spiked control, probably due to the remarkable increase in relative abundance of few taxa. The influence of PAH pollution on bacterial diversities seemed inconsistent as Dos Santos et al. (2011) observed an increase of bacterial richness for sediments after adding oil which is a complex mixture containing different PAH compounds. However, bacterial species diversity in soil or sediments is often extremely high (Curtis et al., 2002; Gans et al., 2005; Hong et al., 2006), only the relatively dominant populations are likely to be detected and the estimation of the bacterial richness is frequently biased by the amount of sequences (see Figure A7 in Appendix) or the algorithm. Populations that decreased in abundance after PAH pollution might be useful indicators of adverse effects of PAH pollutants. In the present study, bacteria belonging to *Conexibacteria*, *Acidobacteria* Gp1, *Myxobacterium* were numerically reduced in the phenanthrene-polluted soils. Among these taxa, only *Acidobacteria* Gp1 was previously reported to be affected by phenanthrene in soil (Sipila et al., 2008). Bacteria belonging to *Solirubrobacter*, *Conexibacter*, *Myxobacterium*, and *Acidobacteria* GP1 prevail in soils but are difficult to be cultivated under standard lab conditions (Davis et al., 2011).

Several studies suggested an important role of fungi in PAH metabolization by direct degradation, by transport of bacterial degraders to the source of PAH (Kohlmeier et al., 2005; Mollea et al., 2005; Furuno et al., 2010; Hong et al., 2010). However, DGGE analysis of PCR-amplified ITS fragments (Weinert et al., 2009) revealed that the fungal communities were less affected by phenanthrene in the present study, except for Cambisol on day 63 (Table 1). Compared to the Luvisol, the Cambisol has a much higher clay content. High clay contents were found to impact bacterial motility or the rate of substrate diffusion in soil (Raynaud and Leadley, 2004; Long and Or, 2009). The transport of bacterial degraders to phenanthrene (Kohlmeier et al., 2005; Furuno et al., 2010) or vice versa on fungal hyphae, might be more important in the Cambisol than in the Luvisol. However, the different response of fungal community to phenanthrene spiking might be also related to the differences in fungal community compositions of both soils (data not shown).

In summary, the responses of soil indigenous bacteria to phenanthrene depended on soil type, exposure time and taxonomic group. In relation to the abundance and diversity of enriched *PAH-RHD*α genes and the relative abundance of enriched taxa in the corresponding control soil, possible synergistic interactions of bacteria belonging to *Sphingomonadaceae* in degrading phenanthrene were suggested. In response to phenanthrene pollution of previously unpolluted soils, numerous OTUs with decreased abundance were identified.

## Conflict of Interest Statement

The authors declare that the research was conducted in the absence of any commercial or financial relationships that could be construed as a potential conflict of interest.

## Footnotes

^1^http://rdp.cme.msu.edu/

^2^http://blast.ncbi.nlm.nih.gov

^3^http://pyro.cme.msu.edu/pyro/help.jsp

^4^http://www.perl.org/

^5^http://www.r-project.org/

## Appendix

**Table A1:** **Primers used in this study**.

Taxonic group	Primer pair	Primer	Sequences (5^′^–3^′^)	Reference
*Bacteria*	F984/R1378	F984	GC clamp-AACGCGAAGAACCTTAC	Heuer et al. (1997)
		R1378	CGGTGTGTACAAGGCCCGGGAACG	
*Alphaproteobacteria*	F203/R1492	F203α	CCGCATACGCCCTACGGGGGAAAGATTTAT	Gomes et al. (2001)
		R1492	TACGGYTACCTTGTTACGACTT	
*Betaproteobacteria*	F948/R1492	F948β	CGCACAAGCGGTGGATGA	Gomes et al. (2001)
*Actinobacteria*	F243/R1378	F243	GGATGAGCCCGCGGCCTA	Heuer et al. (1997)
*Pseudomonas*	gacA-1F/gacA2R	gacA-1F	TGATTAGGGTGYTAGTDGTCGA	Costa et al. (2007)
		gacA2R	MGYCARYTCVACRTCRCTGSTGAT	
	gacA-1FGC/gacA2R	gacA-1FGC	GC clamp-GATTAGGGTGCTAGTGGTCGA	
		gacA2R	GGTTTTCGGTGACAGGCA	
*Fungi*	ITS1F/ITS4	ITS1F	CTTGGTCATTTAGAGGAAGTAA	Weinert et al. (2009)
		ITS4	TCCTCCGCTTATTGATATGC	
	ITS1FGC/ITS2	ITS2	GCTGCGTTCTTCATCGATGC	

*The GC clamp was CGCCCGGGGCGCGCCCCGGGCGGGGCGGGGGCACGGGGGG*.

**Table A2:** **Number of taxa detected**.

	T63CA ((9816)^a^	T63CP (5084)	T63LA (13261)	T63LP (5893)	Total
Phylum	18 (90.8^b^)	13 (94.7)	19 (89.7)	14 (93.3)	21 (91.6)
Class	41 (85.9)	38 (91.6)	51 (84.9)	40 (90.9)	51 (87.5)
Order	47 (60.3)	43 (76.9)	52 (57.2)	46 (76.5)	57 (64.6)
Family	89 (50.8)	79 (72)	100 (48.6)	82 (71)	111(56.8)
Genus	193(48.7)	157(60.6)	210 (49.4)	160 (58.1)	271 (52.5)

*^a^Total number of sequences acquired for the treatment; ^b^percent of sequences which could be classified by Naïve Bayesian classifier at >80% confidence. T63, 63 days after phenanthrene spiking; CA: Cambisol control, CP: phenanthrene-spiked Cambisol, LA: Luvisol control, LP: phenanthrene-spiked Luvisol*.

**Table A3:** **Average of observed OTUs at four different sequences similarities**.

	T63CA	T63CP	T63LA	T63LP
97%	94.6 ± 0.7^a^	81.4 ± 4.7^c^	95.4 ± 0.2^a^	88.6 ± 2.1^b^
90%	83 ± 2^a^	59.9 ± 6^b^	83.8 ± 0.6^a^	67 ± 3.1^b^
80%	54.8 ± 1^a^	39.6 ± 4.8^b^	56.4 ± 0.7^a^	44.9 ± 0.5^b^
70%	28.7 ± 0.4^a^	23 ± 2^b^	30 ± 0.7^a^	24.5 ± 1.3^b^

*Average of observed OTUs was acquired by sampling 100 sequences for each replicate (rarefaction analysis). For each similarity level, treatments with different letters showed significant difference of average observed OTUs (*p* < 0.05 by Tukey HSD test). T63, 63 days after phenanthrene spiking; CA: Cambisol control, CP: phenanthrene-spiked Cambisol, LA: Luvisol control, LP: phenanthrene-spiked Luvisol*.

**Table A4:** **Number of OTUs (>97% similarity) significantly (unadjusted *p* < 0.05) enriched in phenanthrene-spiked soils**.

Phylum	Cambisol	Luvisol	Shared^a^	Total
*Acidobacteria*	1	0	0	1
*Actinobacteria*	3	2	1	4
*Proteobacteria*	44	66	29	81
*Bacteroidetes*	1	0	0	1
Total	49	69	30	88

*^a^Number of OTUs significantly enriched in both phenanthrene-spiked soils*.

## References

[B1] Bell T. Newman J. A. Silverman B. W. Turner S. L. Lilley A. K. (2005). The contribution of species richness and composition to bacterial services. Nature 436, 1157–1160 10.1038/nature03437 16121181

[B2] Brown P. J. Long S. M. Spurgeon D. J. Svendsen C. Hankard P. K. (2004). Toxicological and biochemical responses of the earthworm *Lumbricus rubellus* to pyrene, a non-carcinogenic polycyclic aromatic hydrocarbon. Chemosphere 57, 1675–1681 10.1016/j.chemosphere.2004.05.041 15519413

[B3] Cebron A. Louvel B. Faure P. France-Lanord C. Chen Y. Murrell J. C. Leyval C. (2011). Root exudates modify bacterial diversity of phenanthrene degraders in PAH-polluted soil but not phenanthrene degradation rates. Environ. Microbiol. 13, 722–736 10.1111/j.1462-2920.2010.02376.x 21087382

[B4] Costa R. Gomes N. C. M. Krögerrecklenfort E. Opelt K. Berg G. Smalla K. (2007). Pseudomonas community structure and antagonistic potential in the rhizosphere: insights gained by combining phylogenetic and functional gene-based analyses. Environ. Microbiol. 9, 2260–2273 10.1111/j.1462-2920.2007.01340.x 17686023

[B5] Curtis T. P. Sloan W. T. Scannell J. W. (2002). Estimating prokaryotic diversity and its limits. Proc. Natl. Acad. Sci. U.S.A. 99, 10494–10499 10.1073/pnas.142680199 12097644 PMC124953

[B6] Davis K. E. Sangwan P. Janssen P. H. (2011). *Acidobacteria*, *Rubrobacteridae* and *Chloroflexi* are abundant among very slow-growing and mini-colony-forming soil bacteria. Environ. Microbiol. 13, 798–805 10.1111/j.1462-2920.2010.02384.x 21108723

[B7] Dejonghe W. Berteloot E. Goris J. Boon N. Crul K. Maertens S. Hofte M. De Vos P. Verstraete W. Top E. M. (2003). Synergistic degradation of linuron by a bacterial consortium and isolation of a single linuron-degrading Variovorax strain. Appl. Environ. Microbiol. 69, 1532–1541 10.1128/AEM.69.3.1532-1541.2003 12620840 PMC150106

[B8] Dennis J. J. Zylstra G. J. (2004). Complete sequence and genetic organization of pDTG1, the 83 kilobase naphthalene degradation plasmid from *Pseudomonas putida* strain NCIB 9816-4. J. Mol. Biol. 341, 753–768 10.1016/j.jmb.2004.06.034 15288784

[B9] Ding G. C. Heuer H. He Z. L. Xie J. P. Zhou J. Z. Smalla K. (2012). More functional genes and convergent overall functional patterns detected by GeoChip in phenanthrene spiked soils. FEMS Microbiol. Ecol. 10.1111/j.1574-6941.2012.01413.x 22587620

[B10] Ding G. C. Heuer H. Zühlke S. Spiteller M. Pronk G. J. Heister K. Kögel-Knabner I. Smalla K. (2010). Soil type-dependent responses to phenanthrene as revealed by determining the diversity and abundance of polycyclic aromatic hydrocarbon ring-hydroxylating dioxygenase genes by using a novel PCR detection system. Appl. Environ. Microbiol. 76, 4765–4771 10.1128/AEM.02083-09 20495045 PMC2901735

[B11] Dos Santos H. F. Cury J. C. Do Carmo F. L. Dos Santos A. L. Tiedje J. Van Elsas J. D. Rosado A. S. Peixoto R. S. (2011). Mangrove bacterial diversity and the impact of oil contamination revealed by pyrosequencing: bacterial proxies for oil pollution. PLoS ONE 6, e16943 10.1371/journal.pone.0016943 21399677 PMC3047533

[B12] Edlund A. Jansson J. K. (2008). Use of bromodeoxyuridine immunocapture to identify psychrotolerant phenanthrene-degrading bacteria in phenanthrene-enriched polluted Baltic Sea sediments. FEMS Microbiol. Ecol. 65, 513–525 10.1111/j.1574-6941.2008.00513.x 18616589

[B13] Eom I. C. Rast C. Veber A. M. Vasseur P. (2007). Ecotoxicity of a polycyclic aromatic hydrocarbon (PAH)-contaminated soil. Ecotoxicol. Environ. Saf. 67, 190–205 10.1016/j.ecoenv.2006.12.020 17382389

[B14] Flocco C. G. Gomes N. C. M. Mac C. W. Smalla K. (2009). Occurrence and diversity of naphthalene dioxygenase genes in soil microbial communities from the Maritime Antarctic. Environ. Microbiol. 11, 700–714 10.1111/j.1462-2920.2008.01858.x 19278452

[B15] Furuno S. Pazolt K. Rabe C. Neu T. R. Harms H. Wick L. Y. (2010). Fungal mycelia allow chemotactic dispersal of polycyclic aromatic hydrocarbon-degrading bacteria in water-unsaturated systems. Environ. Microbiol. 12, 1391–1398 19691501 10.1111/j.1462-2920.2009.02022.x

[B16] Gans J. Wolinsky M. Dunbar J. (2005). Computational improvements reveal great bacterial diversity and high metal toxicity in soil. Science 309, 1387–1390 10.1126/science.1112665 16123304

[B17] Giovannoni S. (2004). Evolutionary biology: oceans of bacteria. Nature 430, 515–516 10.1038/430515a 15282590

[B18] Gomes N. C. M. Borges L. R. Paranhos R. Pinto F. N. Krögerrecklenfort E. Mendonca-Hagler L. C. Smalla K. (2007). Diversity of *ndo* genes in mangrove sediments exposed to different sources of polycyclic aromatic hydrocarbon pollution. Appl. Environ. Microbiol. 73, 7392–7399 10.1128/AEM.01099-07 17905873 PMC2168229

[B19] Gomes N. C. M. Borges L. R. Paranhos R. Pinto F. N. Mendonca-Hagler L. C. S. Smalla K. (2008). Exploring the diversity of bacterial communities in sediments of urban mangrove forests. FEMS Microbiol. Ecol. 66, 96–109 10.1111/j.1574-6941.2008.00519.x 18537833

[B20] Gomes N. C. M. Heuer H. Schönfeld J. Costa R. Mendonca-Hagler L. Smalla K. (2001). Bacterial diversity of the rhizosphere of maize (*Zea mays*) grown in tropical soil studied by temperature gradient gel electrophoresis. Plant Soil 232, 167–180 10.1023/A:1010350406708

[B21] Gomes N. C. M. Kosheleva I. A. Abraham W. R. Smalla K. (2005). Effects of the inoculant strain *Pseudomonas putida* KT2442 (pNF142) and of naphthalene contamination on the soil bacterial community. FEMS Microbiol. Ecol. 54, 21–33 10.1016/j.femsec.2005.02.005 16329969

[B22] Gouy M. Guindon S. Gascuel O. (2010). Seaview version 4: a multiplatform graphical user interface for sequence alignment and phylogenetic tree building. Mol. Biol. Evol. 27, 221–224 10.1093/molbev/msp259 19854763

[B23] Gray N. D. Sherry A. Grant R. J. Rowan A. K. Hubert C. R. Callbeck C. M. Aitken C. M. Jones D. M. Adams J. J. Larter S. R. Head I. M. (2011). The quantitative significance of *Syntrophaceae* and syntrophic partnerships in methanogenic degradation of crude oil alkanes. Environ. Microbiol. 13, 2957–2975 10.1111/j.1462-2920.2011.02570.x 21914097 PMC3258425

[B24] Guo C. L. Dang Z. Wong Y. S. Tam N. F. (2010). Biodegradation ability and dioxgenase genes of PAH-degrading *Sphingomonas* and *Mycobacterium* strains isolated from mangrove sediments. Int. Biodeterior. Biodegradation 64, 419–426 10.1016/j.ibiod.2010.04.008

[B25] Han M. V. Zmasek C. M. (2009). phyloXML: XML for evolutionary biology and comparative genomics. BMC Bioinformatics 10, 356 10.1186/1471-2105-10-356 19860910 PMC2774328

[B26] Head I. M. Jones D. M. Roling W. F. (2006). Marine microorganisms make a meal of oil. Nat. Rev. Microbiol. 4, 173–182 10.1038/nrmicro1348 16489346

[B27] Heinaru E. Vedler E. Jutkina J. Aava M. Heinaru A. (2009). Conjugal transfer and mobilization capacity of the completely sequenced naphthalene plasmid pNAH20 from multiplasmid strain *Pseudomonas fluorescens* PC20. FEMS Microbiol. Ecol. 70, 563–574 10.1111/j.1574-6941.2009.00763.x 19744238

[B28] Heuer H. Krsek M. Baker P. Smalla K. Wellington E. M. (1997). Analysis of actinomycete communities by specific amplification of genes encoding 16S rRNA and gel-electrophoretic separation in denaturing gradients. Appl. Environ. Microbiol. 63, 3233–3241 9251210 10.1128/aem.63.8.3233-3241.1997 PMC168621

[B29] Heuer H. Wieland J. Schönwälder A. Gomes N. C. M. Smalla K. (2001). “Bacterial community profiling using DGGE or TGGE analysis,” in Environmental Molecular Microbiology: Protocols and Applications, ed. Rouchelle I. P. (Wymondham: Horizon Scientific Press), 177–190

[B30] Hickey W. J. Chen S. Zhao J. (2012). The phn island: a new genomic island encoding catabolism of polynuclear aromatic hydrocarbons. Front. Microbiol. 3:125 10.3389/fmicb.2012.00125 22493593 PMC3318190

[B31] Hong J. W. Park J. Y. Gadd G. M. (2010). Pyrene degradation and copper and zinc uptake by *Fusarium solani* and *Hypocrea lixii* isolated from petrol station soil. J. Appl. Microbiol. 108, 2030–2040 10.1111/j.1365-2672.2009.04504.x 19922595

[B32] Hong S. H. Bunge J. Jeon S. O. Epstein S. S. (2006). Predicting microbial species richness. Proc. Natl. Acad. Sci. U.S.A. 103, 117–122 10.1073/pnas.0508784103 16368757 PMC1324986

[B33] Hothorn T. Bretz F. Westfall P. (2008). Simultaneous inference in general parametric models. Biom. J. 50, 346–363 10.1002/bimj.200810425 18481363

[B34] Jeon C. O. Park M. Ro H. S. Park W. Madsen E. L. (2006). The naphthalene catabolic (nag) genes of *Polaromonas naphthalenivorans* CJ2: evolutionary implications for two gene clusters and novel regulatory control. Appl. Environ. Microbiol. 72, 1086–1095 10.1128/AEM.72.2.1086-1095.2006 16461653 PMC1392936

[B35] Jeon C. O. Park W. Padmanabhan P. Derito C. Snape J. R. Madsen E. L. (2003). Discovery of a bacterium, with distinctive dioxygenase, that is responsible for in situ biodegradation in contaminated sediment. Proc. Natl. Acad. Sci. U.S.A. 100, 13591–13596 10.1073/pnas.1735529100 14597712 PMC263858

[B36] Johnsen A. R. Schmidt S. Hybholt T. K. Henriksen S. Jacobsen C. S. Andersen O. (2007). Strong impact on the polycyclic aromatic hydrocarbon (PAH)-degrading community of a PAH-polluted soil but marginal effect on PAH degradation when priming with bioremediated soil dominated by mycobacteria. Appl. Environ. Microbiol. 73, 1474–1480 10.1128/AEM.02236-06 17209064 PMC1828760

[B37] Johnsen A. R. Wick L. Y. Harms H. (2005). Principles of microbial PAH-degradation in soil. Environ. Pollut. 133, 71–84 10.1016/j.envpol.2004.04.015 15327858

[B38] Jones M. D. Crandell D. W. Singleton D. R. Aitken M. D. (2011). Stable-isotope probing of the polycyclic aromatic hydrocarbon-degrading bacterial guild in a contaminated soil. Environ. Microbiol. 13, 2623–2632 10.1111/j.1462-2920.2011.02501.x 21564459 PMC4755288

[B39] Keck J. Sims R. C. Coover M. Park K. Symons B. (1989). Evidence for cooxidation of polynuclear aromatic-hydrocarbons in soil. Water Res. 23, 1467–1476 10.1016/0043-1354(89)90110-3

[B40] Kobayashi T. Murai Y. Tatsumi K. Iimura Y. (2009). Biodegradation of polycyclic aromatic hydrocarbons by *Sphingomonas* sp. enhanced by water-extractable organic matter from manure compost. Sci. Total Environ. 407, 5805–5810 10.1016/j.scitotenv.2009.06.041 19660784

[B41] Kohlmeier S. Smits T. H. Ford R. M. Keel C. Harms H. Wick L. Y. (2005). Taking the fungal highway: mobilization of pollutant-degrading bacteria by fungi. Environ. Sci. Technol. 39, 4640–4646 10.1021/es047979z 16047804

[B42] Kropf S. Heuer H. Grüning M. Smalla K. (2004). Significance test for comparing complex microbial community fingerprints using pairwise similarity measures. J. Microbiol. Methods 57, 187–195 10.1016/j.mimet.2004.01.002 15063059

[B43] Laurie A. D. Lloyd-Jones G. (1999). The phn genes of *Burkholderia* sp. strain RP007 constitute a divergent gene cluster for polycyclic aromatic hydrocarbon catabolism. J. Bacteriol. 181, 531–540 9882667 10.1128/jb.181.2.531-540.1999 PMC93407

[B44] Leys N. M. Ryngaert A. Bastiaens L. Wattiau P. Top E. M. Verstraete W. Springael D. (2005). Occurrence and community composition of fast-growing *Mycobacterium* in soils contaminated with polycyclic aromatic hydrocarbons. FEMS Microbiol. Ecol. 51, 375–388 10.1016/j.femsec.2004.09.015 16329885

[B45] Li J. H. Yu X. Z. Wu S. C. Wang X. R. Wang S. H. Tam N. F. Wong M. H. (2011). Responses of bioaugmented ryegrass to PAH soil contamination. Int. J. Phytoremediation 13, 441–455 10.1080/15226510903353104 21598775

[B46] Liang Y. Li G. Van Nostrand J. D. He Z. Wu L. Deng Y. Zhang X. Zhou J. (2009). Microarray-based analysis of microbial functional diversity along an oil contamination gradient in oil field. FEMS Microbiol. Ecol. 70, 324–333 10.1111/j.1574-6941.2009.00774.x 19780823

[B47] Long T. Or D. (2009). Dynamics of microbial growth and coexistence on variably saturated rough surfaces. Microb. Ecol. 58, 262–275 10.1007/s00248-009-9510-3 19352771

[B48] Mollea C. Bosco F. Ruggeri B. (2005). Fungal biodegradation of naphthalene: microcosms studies. Chemosphere 60, 636–643 10.1016/j.chemosphere.2005.01.034 15963802

[B49] Ni Chadhain S. M. Norman R. S. Pesce K. V. Kukor J. J. Zylstra G. J. (2006). Microbial dioxygenase gene population shifts during polycyclic aromatic hydrocarbon biodegradation. Appl. Environ. Microbiol. 72, 4078–4087 10.1128/AEM.02969-05 16751518 PMC1489606

[B50] Pruesse E. Quast C. Knittel K. Fuchs B. M. Ludwig W. Peplies J. Glöckner F. O. (2007). SILVA: a comprehensive online resource for quality checked and aligned ribosomal RNA sequence data compatible with ARB. Nucleic Acids Res. 35, 7188–7196 10.1093/nar/gkm864 17947321 PMC2175337

[B51] Pumphrey G. M. Madsen E. L. (2007). Naphthalene metabolism and growth inhibition by naphthalene in *Polaromonas naphthalenivorans* strain CJ2. Microbiology 153, 3730–3738 10.1099/mic.0.2007/010728-0 17975081

[B52] Raynaud X. Leadley P. W. (2004). Soil characteristics play a key role in modeling nutrient competition in plant communities. Ecology 85, 2200–2214 10.1890/03-0817

[B53] Schafer A. N. Snape I. Siciliano S. D. (2009). Influence of liquid water and soil temperature on petroleum hydrocarbon toxicity in Antarctic soil. Environ. Toxicol. Chem. 28, 1409–1415 10.1897/08-434.1 19245286

[B54] Schloss P. D. (2010). The effects of alignment quality, distance calculation method, sequence filtering, and region on the analysis of 16S rRNA gene-based studies. PLoS Comput. Biol. 6, e1000844 10.1371/journal.pcbi.1000844 20628621 PMC2900292

[B55] Schloss P. D. Westcott S. L. Ryabin T. Hall J. R. Hartmann M. Hollister E. B. Lesniewski R. A. Oakley B. B. Parks D. H. Robinson C. J. Sahl J. W. Stres B. Thallinger G. G. Van Horn D. J. Weber C. F. (2009). Introducing mothur: open-source, platform-independent, community-supported software for describing and comparing microbial communities. Appl. Environ. Microbiol. 75, 7537–7541 10.1128/AEM.01541-09 19801464 PMC2786419

[B56] Schuler L. Jouanneau Y. Chadhain S. M. Meyer C. Pouli M. Zylstra G. J. Hols P. Agathos S. N. (2009). Characterization of a ring-hydroxylating dioxygenase from phenanthrene-degrading *Sphingomonas* sp. strain LH128 able to oxidize benz[a]anthracene. Appl. Microbiol. Biotechnol. 83, 465–475 10.1007/s00253-009-1858-2 19172265

[B57] Singleton D. R. Powell S. N. Sangaiah R. Gold A. Ball L. M. Aitken M. D. (2005). Stable-isotope probing of bacteria capable of degrading salicylate, naphthalene, or phenanthrene in a bioreactor treating contaminated soil. Appl. Environ. Microbiol. 71, 1202–1209 10.1128/AEM.71.3.1202-1209.2005 15746319 PMC1065189

[B58] Singleton D. R. Ramirez L. G. Aitken M. D. (2009). Characterization of a polycyclic aromatic hydrocarbon degradation gene cluster in a phenanthrene-degrading *Acidovorax* strain. Appl. Environ. Microbiol. 75, 2613–2620 10.1128/AEM.01955-08 19270134 PMC2681696

[B59] Singleton D. R. Sangaiah R. Gold A. Ball L. M. Aitken M. D. (2006). Identification and quantification of uncultivated Proteobacteria associated with pyrene degradation in a bioreactor treating PAH-contaminated soil. Environ. Microbiol. 8, 1736–1745 10.1111/j.1462-2920.2006.01112.x 16958754

[B60] Sipila T. P. Keskinen A. K. Akerman M. L. Fortelius C. Haahtela K. Yrjala K. (2008). High aromatic ring-cleavage diversity in birch rhizosphere: PAH treatment-specific changes of I.E.3 group extradiol dioxygenases and 16S rRNA bacterial communities in soil. ISME J. 2, 968–981 10.1038/ismej.2008.50 18563190

[B61] Sorensen S. R. Ronen Z. Aamand J. (2002). Growth in coculture stimulates metabolism of the phenylurea herbicide isoproturon by *Sphingomonas* sp strain SRS2. Appl. Environ. Microbiol. 68, 3478–3485 10.1128/AEM.68.7.3478-3485.2002 12089031 PMC126762

[B62] Sota M. Yano H. Ono A. Miyazaki R. Ishii H. Genka H. Top E. M. Tsuda M. (2006). Genomic and functional analysis of the IncP-9 naphthalene-catabolic plasmid NAH7 and its transposon Tn4655 suggests catabolic gene spread by a tyrosine recombinase. J. Bacteriol. 188, 4057–4067 10.1128/JB.00185-06 16707697 PMC1482893

[B63] Sun W. Xie S. Luo C. Cupples A. M. (2010). Direct link between toluene degradation in contaminated-site microcosms and a *Polaromonas* strain. Appl. Environ. Microbiol. 76, 956–959 10.1128/AEM.01364-09 20008173 PMC2813006

[B64] Uyttebroek M. Breugelmans P. Janssen M. Wattiau P. Joffe B. Karlson U. Ortega-Calvo J. J. Bastiaens L. Ryngaert A. Hausner M. Springael D. (2006). Distribution of the *Mycobacterium* community and polycyclic aromatic hydrocarbons (PAHs) among different size fractions of a long-term PAH-contaminated soil. Environ. Microbiol. 8, 836–847 10.1111/j.1462-2920.2005.00970.x 16623741

[B65] Vacca D. J. Bleam W. F. Hickey W. J. (2005). Isolation of soil bacteria adapted to degrade humic acid-sorbed phenanthrene. Appl. Environ. Microbiol. 71, 3797–3805 10.1128/AEM.71.7.3797-3805.2005 16000791 PMC1169045

[B66] Wang Q. Garrity G. M. Tiedje J. M. Cole J. R. (2007). Naïve Bayesian classifier for rapid assignment of rRNA sequences into the new bacterial taxonomy. Appl. Environ. Microbiol. 73, 5261–5267 10.1128/AEM.00062-07 17586664 PMC1950982

[B67] Weinert N. Meincke R. Gottwald C. Heuer H. Gomes N. C. M. Schloter M. Berg G. Smalla K. (2009). Rhizosphere communities of genetically modified zeaxanthin-accumulating potato plants and their parent cultivar differ less than those of different potato cultivars. Appl. Environ. Microbiol. 75, 3859–3865 10.1128/AEM.00414-09 19376893 PMC2698355

[B68] Yagi J. M. Sims D. Brettin T. Bruce D. Madsen E. L. (2009). The genome of *Polaromonas naphthalenivorans* strain CJ2, isolated from coal tar-contaminated sediment, reveals physiological and metabolic versatility and evolution through extensive horizontal gene transfer. Environ. Microbiol. 11, 2253–2270 10.1111/j.1462-2920.2009.01947.x 19453698

[B69] Zhou H. W. Guo C. L. Wong Y. S. Tam N. F. (2006). Genetic diversity of dioxygenase genes in polycyclic aromatic hydrocarbon-degrading bacteria isolated from mangrove sediments. FEMS Microbiol. Lett. 262, 148–157 10.1111/j.1574-6968.2006.00379.x 16923069

